# Neuroprotective and Antiamnesic Effects of *Mitragyna inermis* Willd (Rubiaceae) on Scopolamine-Induced Memory Impairment in Mice

**DOI:** 10.1155/2017/5952897

**Published:** 2017-03-12

**Authors:** David Bougolla Pahaye, Elisabeth Ngo Bum, Germain Sotoing Taïwé, Gwladys Temkou Ngoupaye, Neteydji Sidiki, Fleur Clarisse Okomolo Moto, Nadège Kouemou, Stephanie Jacqueline Kameni Njapdounke, Gisele Nkantchoua, Antoine Kandeda, Jean Pierre Omam Omam, Veronique Mairaira, Josiane Lucie Ojong

**Affiliations:** ^1^Department of Biological Science, Faculty of Sciences, University of Ngaoundéré, P.O. Box 454, Ngaoundéré, Cameroon; ^2^Institute of Mines and Petroleum Industries, University of Maroua, P.O. Box 46, Maroua, Cameroon; ^3^Department of Zoology and Animal Physiology, Faculty of Science, University of Buea, P.O. Box 63, Buea, Cameroon; ^4^Department of Animal Biology, Faculty of Science, University of Dschang, P.O. Box 67, Dschang, Cameroon; ^5^Higher Teachers' Training College, University of Yaoundé I, P.O. Box 47, Yaoundé, Cameroon; ^6^Department of Animal Biology and Physiology, University of Yaoundé I, P.O. Box 812, Yaoundé, Cameroon; ^7^Center of Medical Research, Institute of Medical Research and Medical Plants Studies, P.O. Box 6163, Yaoundé, Cameroon

## Abstract

*Aim*. To assess memory improvement and neuroprotective and antioxidant effects of *Mitragyna inermis* (*M. inermis*) leaf decoction on the central nervous system. *Methodology*. Leaf decoction of *M. inermis* was tested on learning and memory in normal and scopolamine-induced cognitive impairment in mice using memory behavioral tests such as the Morris water maze, object recognition task, and elevated plus maze. Oxidative stress enzymes—catalase, superoxide dismutase, and the thiobarbituric acid reactive substance, a product of lipid peroxidation—were quantified. In each test, mice 18 to 25 g were divided into groups of 5. *Results*. The extract reversed the effects of scopolamine in mice. The extract significantly increased discrimination index in the object recognition task test and inflexion ratio in the elevated plus maze test. The times spent in target quadrant in MWM increased while the transfer latency decreased in mice treated by *M. inermis* at the dose of 196.5 mg/kg. The activity levels of superoxide dismutase and catalase were significantly increased, whereas the thiobarbituric acid reactive substance was significantly decreased after 8 consecutive days of treatment with *M. inermis* at the dose of 393 mg/kg. *Conclusion*. These results suggest that *M. inermis* leaf extract possess potential antiamnesic effects.

## 1. Introduction

Dementia is a syndrome of gradual onset and continuing decline of higher cognitive functioning. It is a common disorder in older persons and becomes more prevalent in each decade of life [1]. The most common cause of dementia is Alzheimer's disease, which is a progressive neurodegenerative disorder associated with loss of neurons in distinct brain areas [2]. Alzheimer's disease is a progressive neurodegenerative disorder characterized by a gradual decline in memory [3]. Cholinergic system has been found to play a role in learning and memory [4]. It has been shown that cholinergic antagonists, scopolamine and atropine, disrupted memory process in various tasks in animals and in human [5–8]. Nootropic agents such as piracetam are being primarily used to improve memory, mood, and behavior [9]. It is not surprising that they are also used to moderate age-related neurodegenerative pathologies such as Alzheimer's and Parkinson's diseases [10, 11]. However, the resulting adverse effects associated with these agents have limited their use [12]. Despite the availability of different approaches for the discovery of therapeutics, natural plant products still remain one of the best sources of new structural types [13]. Moreover, the World Health Organization has estimated that in developing countries, medicinal plants contribute significantly to primary health [13, 14]. Therefore, it is worthwhile to explore the utility of medicinal plants for the treatment of various cognitive disorders. Leaves of *M. inermis* are used in Cameroonian traditional medicine to treat mental diseases. *M. inermis* is a shrub which grows on low alluvial plains and swampy savannah of many countries in West Africa [15]. *M. inermis* is also found in the sub-Saharan Africa [16, 17]. This plant, used in traditional medicine to treat fever, hypertension, epilepsy, and mental illnesses, demonstrated neither strong in vitro toxicity on human cells nor in vivo genotoxicity in mice [18, 19]. *M. inermis* showed considerable antiplasmodial activity [20, 21]. *M. inermis* leaves phytochemical analysis reveals the presence of alkaloids, catechic tannins, flavonoids, polyphenols, saponosides, sterols, and triterpenes [16]. The aim of our study was therefore to demonstrate the neuroprotective and improvement of learning effects and memory of the decoction of leaves of *M. inermis* in normal as well as in scopolamine-induced cognitive deficit in mice. We used the elevated plus-maze test, the object recognition test, and the Morris water maze test to investigate the integrity of the memory, as well as the quantification of oxidative stress enzymes (catalase and superoxide dismutase) and the thiobarbituric acid reactive substance activity (TBARS), a lipid peroxidation product in mice brain.

## 2. Materials and Methods

### 2.1. Animals

Adult Swiss albino mice of both sexes weighing 18–25 g obtained from the National Veterinary Laboratory of Cameroon (Garoua, Cameroon) were used. The animals were housed in standard polypropylene cages, at 25°C, on a 12/12 h light-dark cycle. Animals received food and water ad libitum. Mice were divided in 7 groups of 5 mice for each test with amnesic induction and 6 groups of 5 mice in each test without amnesic induction. All animal experiments were carried out in accordance with the national principles of laboratory animal care (number FWA-IRB00001954).

### 2.2. The Plant Material, Extraction, and Doses

Fresh leaves of *M. inermis* were collected in the morning during dry season, at Yagoua in the Far North Region of Cameroon. The botanical identification was performed at the National Herbarium of Yaoundé. A voucher specimen was deposited at the Yaoundé Herbarium on the number 8886/SRFcam. The leaves were dried in the shade and were powdered. 10 g of the powder was boiled in 50 mL of distilled water for 20 min. The supernatant was collected, cooled, and filtered (15 mL). After evaporation, 0.59 g of dry extract was obtained (yield: 5.9%).

The doses are obtained from the dose used by the traditional practitioner and the number is obtained from calculation, knowing that the dose of administration is 10 mL/kg.

Volume after filtration: *V* = 15 mL; mass after evaporation: *m* = 0.59.

Initial concentration: Ci = m/V.

Ci = 0.0393 g/mL = 39.3 mg/mL.

Ci = dose/dose of administration.

Volume of administration = 10 mL/kg.

Dose = Ci × dose of administration.

Dose = 393 mg/kg = D1; D1 is the dose used by the traditional practitioner.

D2 = D1/2 = 196.5 mg/kg, D2 is obtained after dilution at 1/2.

D3 = D1/4 = 98.25 mg/kg, D3 is obtained after dilution at 1/4.

D4 = D1/10 = 39.3 mg/kg, D4 is obtained after dilution at 1/10.

### 2.3. Drug and Chemicals

Piracetam (Nootropil (R), UCB S.A., Bruxelles, Belgique) and scopolamine (Pemason Hyoscine(R), Jiangsu Huayang Pharmaceutical Co., China) were procured from the local pharmacies of Ngaoundere, Cameroon.

### 2.4. Experimental Design

Swiss albino mice (18–25 g) of both sexes were used. In each scopolamine-induced amnesia test, mice were divided into 7 groups (*n* = 5) and treated each day for 8 days as follows: group I (scopolamine-treated group) received distilled water (10 mL/kg/day, p.o.) + scopolamine (0.4 mg/kg, i.p.); group II received *M. inermis* (39.3 mg/kg, p.o.) + scopolamine (0.4 mg/kg, i.p.); group III received *M. inermis* (98.25 mg/kg, p.o.) + scopolamine (0.4 mg/kg, i.p.); group IV received *M. inermis* (196.5 mg/kg, p.o) + scopolamine (0.4 mg/kg, i.p.); and group V received *M. inermis* (393 mg/kg, p.o) + scopolamine (0.4 mg/kg, i.p.). Group VI was treated with piracetam (200 mg/kg, p.o.) + scopolamine (0.4 mg/kg, i.p.) and group VII (normal control) received distilled water (10 mL/kg/day, p.o.) + distilled water (10 mL/kg/day, p.o.).

In the elevated plus maze and object recognition task tests, scopolamine (0.4 mg/kg, i.p.) was given to the different groups only on the 8th day, 30 minutes after the respective treatments, to induce cognitive deficit in mice.

In the Morris water maze test, all the administrations were done on the 4th day of the test as follows: group I (scopolamine-treated group) received distilled water (10 mL/kg/day, p.o.); group II received *M. inermis* (39.3 mg/kg, p.o.); group III received *M. inermis* (98.25 mg/kg, p.o.); group IV received *M. inermis* (196.5 mg/kg, p.o); group V received *M. inermis* (393 mg/kg, p.o); group VI was treated with piracetam (200 mg/kg, p.o.); and group VII (normal control) received distilled water (10 mL/kg, p.o.). 30 minutes after the different administration, scopolamine (1 mg/kg, i.p.) was given to each group. Only group VII (normal control) did not receive scopolamine.

In each test without scopolamine, mice were divided into 6 groups (*n* = 5) and treated each day for 8 days as follows: group 1 (normal control) received distilled water (10 mL/kg/day, p.o.); groups 2, 3, 4, and 5 received *M. inermis* at the doses of 39.3, 98.25, 196.5, and 393 mg/kg, p.o., respectively; and group 6 received piracetam (200 mg/kg, p.o.).

Groups I, II, III, IV, V, VI, and VII were used to evaluate the effect on learning and memory in scopolamine-induced cognitive impairment in mice, and to evaluate the effect on learning and memory in normal mice, we rather used groups 1, 2, 3, 4, 5, and 6.

### 2.5. Behavioral Studies

#### 2.5.1. Elevated Plus Maze (EPM) Test

Although the EPM is widely used to induce anxiety [22], now it is also used to evaluate memory [23].

The EPM served as the exteroceptive behavioral model (wherein the stimulus existed outside the body) to evaluate learning and memory in mice [24].

The apparatus consisted of two open arms (25 cm × 5 cm) and two enclosed arms (25 cm × 5 cm × 12 cm). The arms extended from a central platform (5 cm × 5 cm) and the maze was elevated to a height of 40 cm from the floor. On the 8th day, after the last treatment, each mouse was placed at the end of an open arm, facing away from the central platform. The transfer latency (TL), the time taken by mouse with all its four legs to move into one of the enclosed arms, was recorded as L0. If the animal did not enter into one of the enclosed arms within 90 s, it was gently pushed into one of the two enclosed arms and the TL was assigned as 90 s. The mouse was allowed to explore the maze for another 2 minutes and then returned to its home cage. The retention of this learned task was examined 24 h after the 8th day trial (on 9th day) and the TL was recorded as L1. The effect on TL was expressed by inflexion ratio (IR). IR was calculated using the formula: *IR* = (L0 − L1)/L1 [24–26]. TL on the 8th day (L0) reflected learning behavior of animals, whereas on the 9th day, TL (L1) reflected retention of learning behavior.

#### 2.5.2. Object Recognition Test (ORT)

Exploration of novelty in an open-field has been extensively exploited in neuroscience studies of behavior and brain functions in rats and mice, and it is used in the study of memory [27, 28].

The apparatus consisted of a plywood box as used by De Lima et al. with modification (40 × 40 × 30 cm) [29]. The object to be discriminated was made of plywood in two different shapes of 8 cm height. On the day before test, mice were allowed to explore the box (without any object) for 2 minutes. On the day of test in the first trial (T1), two identical objects were presented in two opposite corners of the box, and the time taken by each mouse to explore the objects was recorded. Exploration was considered as directing the nose at a distance less than 2 cm to the object and/or touching with nose. During the second trial (T2, 24 h after T1), a new object replaced one of the objects presented in T1, and mice were left individually in the box for 5 minutes. The time spent for exploring the familiar (F) and the new (N) object was recorded separately, and discrimination index (DI) was calculated as (N − F)/(N + F). Care was taken to avoid place preference and the influence of olfactory stimuli by randomly changing the position of the two objects during T2 and cleaning the apparatus with hydrogen peroxide. The first trial (T1) was conducted 60 minutes after the last treatment on the 8th day. The second trial (T2) was done 24 hours after (T1) [26, 30].

#### 2.5.3. The Morris Water Maze (MWM) Test

The MWM is a white circular pool (100 cm in diameter and 45 cm in height) with a featureless inner surface. The circular pool was filled with water in which 500 mL of milk had been mixed to a height of 30 cm (20 ± 1°C). The pool was divided into four quadrants of equal area. A white platform (6 cm in diameter and 29 cm in height) was centered in one of the four quadrants of the pool and submerged 1 cm below the water surface so that it was invisible at water level. In the water maze experiments, the day prior to the experiment was dedicated to swim training for 60 s in the absence of the platform. In the following days, the mice were given two trial sessions each day for 4 consecutive days. During each trial, the time taken to swim to the platform (escape latency) was recorded. This parameter was averaged for each session of trials and for each mouse. Once the mouse located the platform, it was permitted to remain on it for 10 s. If the mouse did not locate the platform within 120 s, it was placed on the platform for 10 s and then removed from the pool by the experimenter (trial 1). The mouse was given second trial (trial 2) with an intertrial interval of 20 min for 4 consecutive days [31]. In the 4th training day, escape latency (EL) for scopolamine-treated group was compared to the other groups.

Twenty-four hours after the last training session, a probe trial was performed, after removing the platform, to evaluate memory [32].

### 2.6. Biochemical Assays

#### 2.6.1. Supernatant Preparation

Immediately after the test (8th day), the animals were sacrificed and the brain of each animal was carefully isolated. Whole brain samples were rinsed with ice cold normal saline (0.9% NaCl). The tissues were weighed and 20 mg tissue/mL homogenate of brain samples was prepared by homogenizing in phosphate buffer (pH 7.4). The homogenates were centrifuged at 10,000 rev for 10 min at 4°C, and the resulting supernatant was used for the estimation of nonenzymatic and enzymatic antioxidants.

#### 2.6.2. Superoxide Dismutase (SOD) Evaluation

Superoxide radical (O_2_^−^) was generated from the photoreduction of riboflavin and was deducted by nitro blue tetrazolium dye (NBT) reduction method. Measurement of superoxide anion scavenging activity was performed based on the method described by Winterbourne et al., [33]. In 100 *μ*L of supernatant, 700 *μ*L of reactive was introduced. The reactive was composed of 1.5 mL of solution of 100 mM Tris/HCl (pH 7.8), 75 mM NBT, 2 *μ*M riboflavin, and 6 mM EDTA. Absorbance of mixture was measured at 560 nm by a spectrometer. All the tests were performed in triplicate and the results averaged. The results were expressed as units/mg protein.

#### 2.6.3. Catalase (CAT) Evaluation

CAT activity was determined by the method of Sinha, [34]. Briefly, 50 *μ*L of the supernatant of brain homogenate was mixed with 750 *μ*L of 0.1 M phosphate buffer (pH 7.5) and 200 *μ*L of H_2_O_2_. The reaction was terminated by the addition of 200 *μ*L of acid reagent (dichromate/acetic acid mixture). All the tubes were heated for 10 minutes and the absorbance was read spectrometrically at 620 nm. CAT activity was expressed in terms of mmol H_2_O_2_/mg protein.

#### 2.6.4. Thiobarbituric Acid Reactive Substance (TBARS) Activity Assay

The degree of lipid peroxidation was established by the estimation of the concentrations of TBARS in brain. The TBARS content was determined as described by Yagi, [35]. In 250 *μ*L of supernatant, 700 *μ*L of 9% phosphoric acid and 250 *μ*L of thiobarbituric acid were introduced. After agitation, the mixture was boiled in bain-marie for 60 min and cooled. Then, 1250 *μ*L of butanol was added and the mixture was agitated by vortex for 20 s and centrifuged at 25°C for 20 min. The absorbance was spectrometrically read at 534 nm. The results were expressed as mmol/mg protein.

## 3. Phytochemical Characterization Tests

Phytochemical characterization tests of *M. inermis* decoction were realized using qualitative and colorimetric methods (Harbone, [36]), for the determination of principal chemical groups implicated in the treatment of central nervous system diseases.

### 3.1. Alkaloid Determination

In 1 mL of the plant extract, some drops of sulfuric acid (2%) were added. Then, some drops of Meyer's reagent were added to mixture. And white precipitate indicated the presence of alkaloids.

### 3.2. Tannin Determination

3 mL of *M. inermis* decoction was mixed to 5 mL of solution of DMSO 1,5%. The mixture was boiled in bain-marie at 70°C in 3 min and filtered. 2 mL of ferric chloride 3% was added to 3 mL of filtrate. The green dark coloration indicated the presence of catechotannins.

### 3.3. Flavonoid Determination

In 1 mL of the decoction, 2 mL of sodium hydroxide 1N was added. An intense yellow color was produced in the plant extract, which became colorless on addition of a few drops of dilute chlorhydric acid which indicates the presence of flavonoids.

### 3.4. Anthraquinone Determination

0,5 mL of *M. inermis* decoction was mixed to 5 mL of diethyl ether. The mixture was agitated for homogenization and then let to rest. After addition of ammonia 10%, red or violet coloration indicated the effective presence of anthraquinones in the extract.

## 4. Statistical Analysis

The data were expressed as mean ± SD. Statistical significances of differences among treatments were determined by use of one-way analysis of variance (ANOVA), followed by Dunnett's bilateral comparisons or by Duncan's test. Differences were considered significant when *P* < 0.05.

## 5. Results

### 5.1. Phytochemical Characterization Tests

Results of phytochemical characterization tests show the presence of flavonoids, alkaloids, tannins, and anthraquinones in the leaf decoction of *M. inermis*.

### 5.2. Effects of *M. inermis* in EPM

In normal mice, administration of different doses of *M. inermis* for 8 days significantly decreased the TL (*P* < 0.05) and significantly increased the IR compared to normal control group (*P* < 0.001) (Table 1).

Mice that received distilled water and scopolamine (57.2 s) showed a significant increase of TL compared to normal control group (40.2 s) (*P* < 0.001). These mice showed also a significant decrease of the IR compared to normal control group (*P* < 0.001) (Table 1). On the contrary, mice treated with the plant extract showed a significant decrease of TL and a significant increase of IR when compared to scopolamine-treated group. The IR increased in a dose-dependent manner and the maximum was observed at the dose 196.5 mg/kg (*P* < 0.001). At the dose 196.5 mg/kg, the TL was 36.2 ± 2.16 and the IR 0.9. Mice treated with piracetam showed also a significant decrease of TL (*P* < 0.001) and a significant increase of IR (*P* < 0.001) compared to the scopolamine-treated group. The effects of the plant extract at the dose 196.5 mg/kg were greater than the effect of piracetam (Table 2).

### 5.3. Effects of *M. inermis* in ORT

In normal mice, the extract of *M. inermis*, like piracetam, significantly increased the discrimination index and the exploration time of the novel object and decreased the exploration time of the familiar object (*P* < 0.01) (Table 3).

The extract of *M. inermis* significantly increased the discrimination index from 0.08 in the scopolamine-treated group (group I) to 0.54 and 0.61 (*P* < 0.001) in mice treated with *M. inermis* at the doses 98.25 and 196.5 mg/kg, p.o., respectively. The exploration time of the novel object was also significantly increased by *M. inermis* from 21.2 ± 0.6 in the scopolamine-treated group to 38.0 ± 1.6 and 40.2 ± 3.0 in mice treated with *M. inermis* at the doses 98.25 and 196.5 mg/kg, p.o., respectively (*P* < 0.001). The two same doses of *M. inermis* significantly decreased the exploration time of the familiar object (*P* < 0.001). Piracetam significantly increased the discrimination index and the exploration time of the novel object and decreased the exploration time of the familiar object (*P* < 0.001). Here also, the effect of *M. inermis* at the dose 196.5 mg/kg is greater than the effect of piracetam (Table 4).

### 5.4. Effects of *M. inermis* in MWM

After acquisition time in the first three days, the administration of scopolamine in the 4th day resulted in a significant increase of the escape latency compared to normal control group, from 41.2 ± 1.3 s in the normal control group (VII) to 48.2 ± 3.7 s in the scopolamine-treated group of mice (I) (*P* < 0.01). The different doses of *M. inermis* significantly decreased the escape latency induced by scopolamine from 48.2 ± 3.7 s in the scopolamine-treated group to 41.0 ± 1.6 and 32.2 ± 1.9 s when mice were treated with *M. inermis* at the doses 98.25 and 196.5 mg/kg, respectively (*P* < 0.01 and *P* < 0.001). Piracetam (200 mg/kg) also significantly decreased the escape latency to 41.6 ± 2.7 (*P* < 0.01) (Table 5).

In probe trial performed 24 h after the last training session, scopolamine (1 mg/kg, i.p.) significantly decreased the time spent by mice scopolamine-treated group (group I, 50.6 ± 3.0 s) in the target quadrant compared to normal control group (group VII, 65.4 ± 3.21 s) (*P* < 0.01). *M. inermis* significantly increased the time spent in target quadrant. This time increased from 50.6 ± 3.0 s in the mice scopolamine-treated group to 68.4 ± 2.7 in mice treated with *M. inermis* extract at the dose 196.5 mg/kg (*P* < 0.001). Piracetam (200 mg/kg) also significantly increased the time spent in target quadrant (*P* < 0.001) (Table 5).

### 5.5. Effects of *M. inermis* on Antioxidant Enzymes and TBARS Activities

Scopolamine (0.4 mg/kg, i.p.) significantly decreased (*P* < 0.001) CAT and SOD activities of scopolamine-treated groups (group I) compared to normal control groups (group VII). Mice treated with *M. inermis* showed a significant increase in CAT activity. The CAT activity was increased from 58.58 ± 4.65 mmol H_2_O_2_/mg protein in the scopolamine-treated group to 251.44 ± 32.55, 274.36 ± 8.78, and 325 ± 18.89 mmol H_2_O_2_/mg protein in groups treated with *M. inermis* at the doses 98.25, 196.5, and 393 mg/kg, respectively (*P* < 0.001). SOD activity also significantly increased from 0.76 ± 0.23 units/mg protein in scopolamine-treated group to 5.05 ± 0.48, 4.86 ± 0.19, and 6.39 ± 0.42 units/mg protein in groups treated with the same doses of *M. inermis* (*P* < 0.001). CAT and SOD activities were significantly increased by piracetam (*P* < 0.001) (Table 6).

Scopolamine induced a significant increase in the TBARS concentration in the scopolamine-treated group (group I, 1.32 ± 0.21 mmol/mg protein) compared to the normal group (group VII, 0.63 ± 0.03 mmol/mg protein) (*P* < 0.01). The doses 98.25, 196.5, and 393 mg/kg of *M. inermis* significantly decreased the TBARS concentration to 0.64 ± 0.10, 0.62 ± 0.06, and 0.59 ± 0.01 mmol/mg protein, respectively (*P* < 0.01). Piracetam also induced a significant increase in the TBARS activity (*P* < 0.01) (Table 6).

## 6. Discussion

Scopolamine significantly decreased IR and significantly increased TL in mice, indicating impairment of learning and memory in the EPM. That result was obtained by other authors [37–39], since scopolamine is a muscarinic cholinergic receptor antagonist which causes memory impairments in rodents especially in the processes of learning acquisition and short-term memory [40–42]. The fact that pretreatment with *M. inermis* for 8 days increased IR and decreased TL indicated the improvement in learning and memory in normal mice (without scopolamine). *M. inermis* also increased IR and decreased TL scopolamine-treated mice, indicating the learning and memory processes protection [43].

In ORT, the increase of the DI and novel object exploration time in mice treated with *M. inermis* suggested improvement of learning and memory [44–46]. Scopolamine significantly decreased the discrimination index, indicating impairment of learning and memory. This effect was reversed by *M. inermis* since *M. inermis* significantly increased DI and novel object exploration time, indicating that *M. inermis* possesses memory-enhancing activity in view of its facilitatory effect on remembering the original object [47]. These results showed that *M. inermis* may play a nootropic role in mice brain. Nootropic agents or cognitive enhancers are reported to improve mental functions such as cognition, memory, or attention [48].

MWM test (Table 7) showed increase in escape latency in scopolamine-treated group indicating memory impairment induced by the scopolamine [49–51]. *M. inermis* significantly reversed this effect by decreasing the escape latency time prolonged by scopolamine, suggesting also the remembering on the platform by these mice [49, 50]. MWM is used as a device to investigate spatial learning and memory and for the validation of rodent models for neurocognitive disorders and the evaluation of possible neurocognitive treatments [52].

In the probe trial, the *M. inermis*-treated mice showed a significant longer stay in the platform quadrant. Consequently, these results suggest that *M. inermis* improves long-term and reference memory impairments induced by scopolamine [50, 51, 53, 54].

SOD is an important enzyme in living cells for maintaining normal physiological conditions and coping with oxidative stress [55]. Decreased SOD serum levels represent consumption of SOD during increased oxygen free radical activity [56]. As *M. inermis* strongly reversed the decrease of CAT and SOD induced by scopolamine in the brain and increased the level of CAT and SOD in the brain, it was suggested that *M. inermis* possess antioxidant activities. Coupling to that, TBARS is an end product of lipid peroxidation [57]. Increased TBARS activity has been shown to be an important marker for in vivo lipid peroxidation in the learning and memory deficient mouse brains [58]. In addition, it has been reported that scopolamine significantly increases TBARS activity in the hippocampus and frontal cortex [59–62]. These results are in agreement with our findings where brain TBARS activity was increased in the scopolamine-treated mice.

Restraint stress causes significant reduction in CAT, SOD, and GSH levels and increased lipid peroxidation in rat brain [63]. Malondialdehyde (MDA) is one of the oxidative damage products of lipid peroxidation. Its presence in tissues indicates oxidative stress [64]. A decrease in the activities of these antioxidant enzymes can lead to an excess availability of O2 and H_2_O_2_, which in turn generate OH, resulting in initiation and propagation of lipid peroxydation [65]. Reversing the plant extract of the increase of TBARS activity induced by scopolamine also suggested the antioxidant activity of the plant. Increase of superoxide dismutase (SOD), catalase (CAT), and glutathione peroxidase (GPx) levels, and reduced glutathione (GSH) and decreased level of lipid peroxidation (TBARS), is an exhibition of significant antioxidant activity [66]. Antioxidant enzymes such as superoxide dismutase (SOD), glutathione peroxidase (GPX), and catalase as well as glutathione reductase (GSH) and ascorbate are involved in the reduction of oxidative stress [67]. Antioxidant enzymes display the reduced activities in the affected brain region of patients of Alzheimer's disease [67].

*M. inermis* has clearly demonstrated neuroprotective properties*. M. inermis* inhibited brain TBARS levels and raised the endogenous antioxidants SOD and CAT suggesting that its neuroprotective effects may be due to antioxidant action and, therefore, might play an effective free radical scavenger and antioxidant.

Phytochemical analysis of *M. inermis* leaf decoction showed the presence of flavonoids, alkaloids, saponosides, polyphenols, catechic tannins, sterols, and triterpenes [16]. Flavonoids can protect the brain by their ability to modulate intracellular signals promoting cellular survival [68]. Flavonoids have antioxidant effects [69–71].

In conclusion, *M. inermis* has learning- and memory-enhancing effect in normal mice and antiamnesic activity in a scopolamine mice model. It exhibited a cognitive-enhancing effect by reversing scopolamine-induced learning and memory deficits and neurotoxicity at least in part through improvement in brain levels of SOD, CAT, and MDA. These results confirm at least in part the common use of *M. inermis* in traditional medicine as a neuroprotective and antiamnesic plant. However, further studies are needed to understand the mechanisms used by *M. inermis* to exercise its effects.

## Figures and Tables

**Table 1:** Effect of *M. inermis* in normal mice in EPM.

Treatments	Transfer latency (seconds)		Inflexion ratio
	8th day	9th day	
H2O	69.6 ± 9.9	63.2 ± 2.2	0.10
39.3	69.6 ± 7.5	43 ± 2.4	0.62^∗∗^
98.25	63.0 ± 6.0	33.4 ± 1.5^∗^	0.89^∗∗∗^
196.5	66.2 ± 6.2	34.4 ± 5.4^∗^	0.92^∗∗∗^
393	61.6 ± 6.3	38.4 ± 3.9^∗^	0.60^∗^
Pira	60.6 ± 3.8	32.6 ± 4.6^∗^	0.86^∗∗∗^

Data are mean ± SD, *n* = 5 per dose. ^∗^*P* < 0.05, ^∗∗^*P* < 0.01, and ^∗∗∗^*P* < 0.001, compared to control group (H2O), one-way ANOVA followed by Dunnett's bilateral comparisons for TL, and Duncan's test for IR. H2O: distilled water, Pira: piracetam 200 mg/kg.

**Table 2:** Effect of *M. inermis* in scopolamine-induced cognitive deficit mice in EPM.

Treatments	Transfer latency (seconds)		Inflexion ratio
	8th day	9th day	
H2O + Scop	62.4 ± 2.6	57.2 ± 1.8^c^	0.09^c^
39.3 + Scop	63.2 ± 7.04	39.0 ± 2.4^∗∗∗^	0.62^∗∗^
98.25 + Scop	65.4 ± 5.12	37.8 ± 2.16^∗∗∗^	0.73^∗∗∗^
196.5 + Scop	68.8 ± 2.96	36.2 ± 2.16^∗∗∗^	0.90^∗∗∗^
393 + Scop	63.6 ± 2.32	46.4 ± 1.92^∗^	0.37^∗^
Pira + Scop	63.4 ± 4.72	38.6 ± 2.08^∗∗∗^	0.64^∗∗∗^
H2O + H2O	69.6 ± 8.32	40.2 ± 1.44^∗∗∗^	0.73^∗∗∗^

Data are mean ± SD, *n* = 5 per dose. ^∗^*P* < 0.05, ^∗∗^*P* < 0.01, and ^∗∗∗^*P* < 0.001, compared to scopolamine-treated group (H2O + Scop), one-way ANOVA followed by Dunnett's bilateral comparisons for TL, and Duncan's test for IR. ^c^*P* < 0.001 compared to normal control group (H2O + H2O), one-way ANOVA followed by Dunnett's bilateral comparisons for TL, and Duncan's test for IR. H2O: distilled water, Pira: piracetam 200 mg/kg, Scop: scopolamine 0.4 mg/kg.

**Table 3:** Effect of *M. inermis* in normal mice in ORT.

Treatments	Exploration time (seconds)			Discrimination index
	Session 1	Session 2		
		N	F	
H2O	36.0 ± 4.5	25.2 ± 1.5	15.0 ± 1.6	0.25
39.3	39.8 ± 3.8	31.8 ± 2.2^∗^	11 ± 1^∗^	0.48^∗^
98.25	39.8 ± 3.7	31.2 ± 3.1^∗^	10.2 ± 0.83^∗^	0.51^∗∗^
196.5	36.2 ± 1.3	33.4 ± 2.6^∗∗^	9.4 ± 0.9^∗∗^	0.56^∗∗∗^
393	38.4 ± 2.4	32.6 ± 2.1^∗∗^	10.6 ± 0.9^∗^	0.51^∗∗^
Pira	34.8 ± 4.2	33.2 ± 1.9^∗∗^	9.6 ± 1.1^∗∗^	0.55^∗∗∗^

Data are mean ± SD, *n* = 5 per dose. ^∗^*P* < 0.05, ^∗∗^*P* < 0.01, and ^∗∗∗^*P* < 0.001, compared to control group (H2O), one-way ANOVA followed by Dunnett's bilateral comparisons for exploration time, and Duncan's test for discrimination index. F: familiar object, H2O: distilled water, N: novel object, Pira: piracetam 200 mg/kg.

**Table 4:** Effect of *M. inermis* in scopolamine-induced cognitive deficit mice in ORT.

Treatments	Exploration time (seconds)			Discrimination index
	Session 1	Session 2		
		N	F	
H2O + Scop	31.4 ± 2.1	21.2 ± 0.64^c^	18 ± 1.2^c^	0.08^c^
39.3 + Scop	38.2 ± 1.8	36.2 ± 3.0^∗∗∗^	13.8 ± 0.7^∗∗^	0.45^∗^
98.25 + Scop	34.8 ± 2.6	38.0 ± 1.6^∗∗∗^	11.4 ± 1.3^∗∗∗^	0.54^∗∗∗^
196.5 + Scop	38.0 ± 3.6	40.2 ± 3.0^∗∗∗^	9.6 ± 1.9^∗∗∗^	0.61^∗∗∗^
393 + Scop	32.0 ± 3.2	32.0 ± 2.8^∗∗∗^	10.8 ± 1.4^∗∗∗^	0.49^∗∗^
Pira + Scop	33.0 ± 2.8	35.4 ± 2.5^∗∗∗^	11.8 ± 1.5^∗∗∗^	0.50^∗∗∗^
H2O + H2O	30.0 ± 1.6	36.0 ± 3.6^∗∗∗^	11.2 ± 1.1^∗∗∗^	0.52^∗∗∗^

Data are mean ± SD, *n* = 5 per dose. ^∗^*P* < 0.05, ^∗∗^*P* < 0.01, and ^∗∗∗^*P* < 0.001, compared to scopolamine-treated group (H2O + Scop), one-way ANOVA followed by Dunnett's bilateral comparisons for exploration time, and Duncan's test for discrimination index. ^c^*P* < 0.001 compared to normal control group (H2O + H2O), one-way ANOVA followed by Dunnett's bilateral comparisons for exploration time, and Duncan's test for discrimination index. F: familiar object, H2O: distilled water, N: novel object, Pira: piracetam 200 mg/kg, Scop: scopolamine 0.4 mg/kg.

**Table 5:** Effect of *M. inermis* in scopolamine-induced cognitive impairment mice in MWM.

Doses	Escape latency (seconds)				Time spent in target quadrant (seconds)
	Day 1	Day 2	Day 3	Day 4	
H2O + Scop	105 ± 7.5	90.4 ± 9.8	65.4 ± 7.4	48.2 ± 3.7^b^	50.6 ± 2.9^b^
39,3 + Scop	108.8 ± 6.8	90.8 ± 10.6	65.6 ± 7.0	44.6 ± 3.4	53.0 ± 3.5
98,25 + Scop	101.8 ± 10.8	95 ± 9.9	65.4 ± 6.9	41.0 ± 1.6^∗∗^	63.2 ± 2.6^∗∗^
196,5 + Scop	104.6 ± 7.5	91.6 ± 7.7	53.2 ± 6.1	32.2 ± 1.9^∗∗∗^	68.4 ± 2.7^∗∗∗^
393 + Scop	98 ± 5.8	86 ± 9.9	50 ± 7.3	39.8 ± 1.3^∗∗∗^	64.8 ± 3.8^∗∗^
Pira + Scop	105.4 ± 8.9	85.4 ± 7.4	53.8 ± 9.6	41.6 ± 2.7^∗∗^	66.0 ± 2.6^∗∗∗^
H2O + H2O	101 ± 7.8	86.2 ± 6.3	62.8 ± 7.7	41.2 ± 1.3^∗∗^	65.4 ± 3.2^∗∗^

Data are mean ± SD, *n* = 5 per dose. ^∗∗^*P* < 0.01, and ^∗∗∗^*P* < 0.001, compared to scopolamine treated group (H2O + Scop), one-way ANOVA followed by Dunnett's bilateral comparisons. ^b^*P* < 0.01 compared to normal control group (H2O + H2O), one-way ANOVA followed by Dunnett's bilateral comparisons. H2O: distilled water, Pira: piracetam 200 mg/kg, Scop: scopolamine 1 mg/kg.

**Table 6:** Chemical evaluation of CAT, SOD, and MDA activities.

Treatments	CAT (mmol H_2_O_2_/mg protein)	SOD (units/mg protein)	TBARS (mmol/mg protein)
H2O + Scop	58.6 ± 4.6^c^	0.8 ± 0.2^c^	1.3 ± 0.2^b^
39.3 + Scop	115.8 ± 1.1^∗^	2.4 ± 0.12^∗^	0.8 ± 0.1^∗^
98.25 + Scop	251.4 ± 32.6^∗∗∗^	5.1 ± 0.5^∗∗^	0.6 ± 0.1^∗∗^
196.5 + Scop	274.4 ± 8.8^∗∗∗^	4.9 ± 0.2^∗∗^	0.6 ± 0.1^∗∗^
393 + Scop	325.0 ± 18.9^∗∗∗^	6.4 ± 0.4^∗∗∗^	0.6 ± 0.1^∗∗^
Pira + Scop	179.2 ± 9.0^∗∗^	4.7 ± 0.3^∗∗^	0.7 ± 0.1^∗∗^
H2O + H2O	244.5 ± 10.9^∗∗∗^	6.7 ± 0.2^∗∗∗^	0.6 ± 0.1^∗∗^

Data are mean ± SEM, *n* = 5 per dose. ^∗^*P* < 0.05, ^∗∗^*P* < 0.01, and ^∗∗∗^*P* < 0.001 as compared with scopolamine-treated group (H2O + Scop), one-way ANOVA followed by Duncan's test; ^b^*P* < 0.01, ^c^*P* < 0.001 compared to the normal control group (H2O + H2O), one-way ANOVA followed by Duncan's test. Pira: piracetam, H2O: distilled water, CAT: catalase, SOD: superoxide dismutase, TBARS: thiobarbituric acid reactive substance activity.

**Table 7:** MWM Test.

*Escape latency day 1*							
Mice/doses	H2O + Scop	39.3 + Scop	98.25 + Scop	196.5 + Scop	393 + Scop	Pira + Scop	H2O + H2O
1	110	112	101	107	89	112	102
2	112	110	91	111	105	100	114
3	109	102	97	109	99	107	94
4	95	118	120	104	97	93	98
5	99	102	100	92	100	115	97
Means	105	108.8	101.8	104.6	98	105.4	101
SD	7.51664819	6.87022561	10.894953	7.50333259	5.83095189	8.96102673	7.81024968

*Escape latency day 2*							
Mice/doses	H2O + Scop	39.3 + Scop	98.25 + Scop	196.5 + Scop	393 + Scop	Pira + Scop	H2O + H2O
1	84	95	102	97	101	79	83
2	90	80	99	100	89	89	95
3	78	102	92	80	78	93	78
4	98	98	103	89	76	90	86
5	102	79	79	92	86	76	89
Means	90.4	90.8	95	91.6	86	85.4	86.2
SD	9.8386991	10.6160256	9.92471662	7.76530746	9.97496867	7.43639698	6.37965516

*Escape latency day 3*							
Mice/doses	H2O + Scop	39.3 + Scop	98.25 + Scop	196.5 + Scop	393 + Scop	Pira + Scop	H2O + H2O
1	70	67	57	62	53	53	62
2	72	65	59	57	50	56	62
3	65	72	72	49	60	67	57
4	53	54	71	47	47	40	76
5	67	70	68	51	40	53	57
Means	65.4	65.6	65.4	53.2	50	53.8	62.8
SD	7.43639698	7.02139587	6.94982014	6.18061486	7.38241153	9.62808392	7.79102047

*Escape latency day 4*							
Mice/doses	H2O + Scop	39.3 + Scop	98.25 + Scop	196.5 + Scop	393 + Scop	Pira + Scop	H2O + H2O
1	43	49	41	31	41	38	43
2	50	46	40	32	38	42	40
3	48	45	43	35	41	40	42
4	53	43	39	30	39	45	41
5	47	40	42	33	40	43	40
Means	48.2	44.6	41	32.2	39.8	41.6	41.2
SD	3.7013511	3.36154726	1.58113883	1.92353841	1.30384048	2.70185122	1.30384048

*Time spent in target quadrant*							
Mice/doses	H2O + Scop	39.3 + Scop	98.25 + Scop	196.5 + Scop	393 + Scop	Pira + Scop	H2O + H2O
1	48	51	61	71	62	65	65
2	50	55	60	68	65	63	67
3	55	49	66	70	68	65	70
4	48	58	64	69	60	67	63
5	52	52	65	64	69	70	62
Means	50.6	53	63.2	68.4	64.8	66	65.4
SD	2.96647939	3.53553391	2.58843582	2.70185122	3.8340579	2.64575131	3.20936131
